# Detection of peste des petits ruminants virus in pneumonic lungs from apparently healthy sheep and goats slaughtered at Al-Hasaheisa slaughterhouse, Gezira state, central Sudan

**DOI:** 10.4102/ojvr.v87i1.1892

**Published:** 2020-12-17

**Authors:** Alaa E.M. Alhussain, Nahid A.S. Abdalla, Sana I. Mohammed, Mihad Hakeem, Ihsan H. Ahmed, Nussieba A. Osman

**Affiliations:** 1 Department of Pathology, Parasitology and Microbiology, College of Veterinary Medicine, Sudan University of Science and Technology (SUST), Khartoum-North, Sudan; 2 Virology Department, Central Veterinary Research Laboratory (CVRL), Soba, Khartoum, Sudan; 3 Epidemiology Department, Central Veterinary Research Laboratory (CVRL), Soba, Khartoum, Sudan; 4 Viral Vaccine Production Department, Central Veterinary Research Laboratory (CVRL), Soba, Khartoum, Sudan

**Keywords:** peste des petits ruminants, PPRV, Sudan, sheep, goats, pneumonic lungs, haemagglutination test

## Abstract

The study aimed to investigate the presence of peste des petits ruminants (PPR) in pneumonic lung tissues from clinically apparently healthy sheep and goats and further demonstrating its prevalence in Gezira state, central Sudan. During March 2019, 99 pneumonic lung samples were collected from apparently healthy sheep (80) and goats (19) from Al-Hasaheisa slaughterhouse located in Al-Hasaheisa locality, Gezira state. Using the haemagglutination (HA) test for the detection of peste des petits ruminants virus (PPRV) antigen, an overall antigenic prevalence of 86.9% was demonstrated in sheep and goats lung tissue homogenate. Of note, the prevalence of PPRV is higher in goats (100%) compared to sheep (83.7%). In this study, the reported increasing prevalence of PPR in central Sudan might be because of insufficient vaccination of animals. The findings of the present study indicated the widespread of PPR amongst sheep and goats in Al-Hasaheisa, Gezira state. Detection of PPRV antigen in the pneumonic lung samples is an indication of exposure of these animals to PPRV or presence of PPR viral infection and demonstrates the role of PPR as the cause of pneumonia in small ruminants. In fact, the circulation of the virus in clinically apparently healthy animals poses a threat for other in-contact susceptible animals and could play a significant role in the spread of the disease.

## Introduction

Peste des petits ruminants (PPR) is a highly contagious and fatal viral disease affecting domestic and wild small ruminants in developing countries (Edo, Deneke & Abdela 2017; OIE 2019). Peste des petits ruminants virus (PPRV) causes high rates of deaths with morbidity and mortality rates ranging from 90% to 100% (Dhar et al. 2002; Singh & Prasad 2008) amongst infected small animals, hence has a major economic effect in livestock, particularly in endemic areas, including the inter-tropical regions of Africa, the Arabian Peninsula, the Middle East and Asia (OIE 2019). Peste des petits ruminants virus also causes high rates of abortion in goats (Abubakar, Ali & Khan 2008).

Peste des petits ruminants is caused by PPRV, classified in the *Small Ruminant Morbillivirus* (*SRMV*) species, in the *Morbillivirus* genus, in the *Paramyxoviridae* family (Gibbs et al. 1979; Maes et al. 2019). Like other Morbillivirus members, PPRV is highly pathogenic for its natural hosts ‘small ruminants’ (Gibbs et al. 1979).

Peste des petits ruminants has been existing in Sudan since the first reported outbreak in 1971 in sheep and goats in three areas in Elgedarif (Elhag Ali 1973), which was at first diagnosed as rinderpest but later confirmed as PPR (Elhag Ali & Taylor 1984). Peste des petits ruminants outbreaks were then reported in goats in Sinnar, central Sudan, during 1971–1972 and in Mieliq in 1972 (Elhag Ali & Taylor 1984). Afterwards, PPR outbreaks were reported in Gezira state during 1990s (El Hassan et al. 1994). After the 2000s, outbreaks of PPR were reported in many parts of Sudan in sheep and goats (Osman 2005; Osman et al. 2018; Saeed et al. 2004, 2010, 2017), camels (Khalafalla et al. 2010; Kwiatek et al. 2011) and Dorcas gazelles (Asil et al. 2019), affecting the economy of the country. In recent years, PPR was demonstrated in lung samples collected from clinically healthy animals that showed lesions on post-mortem (PM) in slaughterhouses in Sudan (Saeed et al. 2017). Sheep, goats and cattle are the main sources of meat production in Sudan. Knowing that PPR is endemic causing significant losses amongst small ruminants, it is important to investigate its presence in the slaughtered animals. The present study aimed to investigate the presence of PPR and demonstrate the prevalence of PPRV antigen in pneumonic lung tissues from clinically apparently healthy sheep and goats slaughtered at Al-Hasaheisa slaughter house, Gezira state, central Sudan.

## Materials and methods

### Study area

**FIGURE 1:**
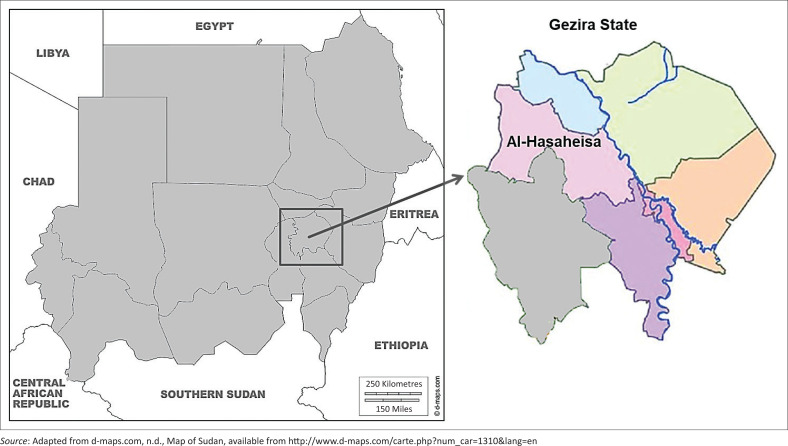
Map of Sudan showing the study area ‘Al-Hasaheisa area in Gezira state, central Sudan’.

The study was conducted in Al-Hasaheisa slaughterhouse located in Al-Hasaheisa locality, Gezira state (central Sudan) (Figure 1). Gezira state has an area of 27 549 square kilometres (km^2^) and is located between the coordinates 14°30’ North and 33°30’ East in central Sudan. Gezira state is composed of seven localities; however, the study was conducted only in Al-Hasaheisa locality in the western area of the state. Lung tissue samples were collected from Al-Hasaheisa abattoir, which is located at the margins of the town. Sheep, goats and cattle were slaughtered daily, except on Monday, in Al-Hasaheisa abattoir.

### Reference virus

The live attenuated PPRV Nigeria 75/1 vaccine strain was obtained as a freeze-dried preparation, dissolved into 0.5 millitres (mL) of sterile distilled water, stored at -20°C till used. The PPR vaccine was the positive control PPRV antigen used in the HA test and kindly provided by the Viral Vaccine Department, Central Veterinary Research Laboratory (CVRL), Soba, Khartoum.

### Sample collection and preparation

During March to April 2019, a total of 99 lung samples with pneumonic lesions were collected from clinically apparently healthy sheep and goats, without considering its different sex, ages and breeds, from Al-Hasaheisa abattoir. These tissues were collected from sheep (80 samples) and goat (19 samples), preserved into sealed plastic bags, frozen at −20 °C.

A 20% (w/v) tissue homogenate containing viral antigen was prepared from sheep and goats lung tissues in phosphate-buffer saline (PBS) (pH 7.2–7.4) supplemented with antibiotics [1 mL of Penicillin (200 international units [IU]/mL), 1 mL of Streptomycin (100 micrograms [*µ*g]/mL), 1 mL of Gentamycin (10 000 *µ*g/mL)] and antifungal [0.5 mL of Fungizone (50 000 IU/mL)] following the standard procedure. The homogenate was distributed into Eppendorf tubes and stored at −20 °C and then used as antigen source for PPRV in the haemagglutination (HA) test.

### Preparation of red blood cells suspension

Whole blood was collected, from healthy non-immunised chickens, in vacutainers containing anticoagulant ‘Alsever’s solution’, red blood cells (RBCs) were washed three times and 0.8% of RBCs suspension was prepared in PBS (pH 6.8) following the standard procedure, the RBCs suspension was used as an indicator in the HA test.

### Haemagglutination test

**FIGURE 2:**
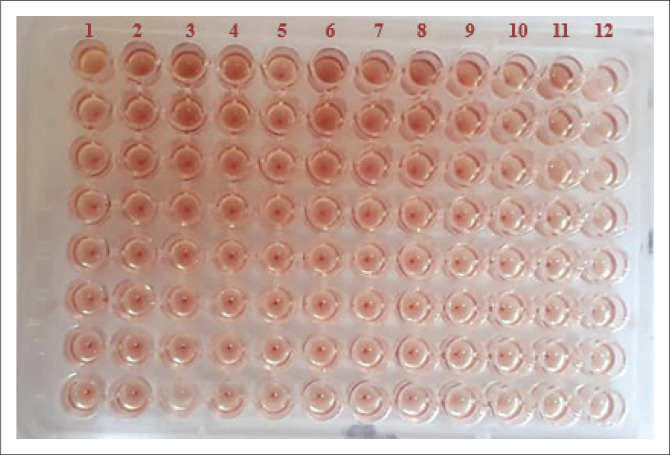
Haemagglutination test plate for detection of peste des petits ruminants virus antigen showing positive haemagglutination result as a diffused sheet or film and negative HA result as a sharp red button of sediment red blood cells. Peste des petits ruminants virus Control (Column 1), antigen samples (Columns 2–11), red blood cells control (Column 12). (Samples are in vertical position).

The HA test was used for identifying and quantifying PPRV antigen in the 20% tissue suspension of sheep and goats lung tissues following the procedure described previously by Ezeibe, Wosu and Erumaka (2004) and Osman (2005) with some modifications for the test conditions. To achieve highest HA titres, the HA test was performed in 96-well V-shaped bottom microtiter HA plates using PBS (pH 6.8) as diluent, chicken RBCs suspension at 0.8% concentration, and the test plates were incubated in a refrigerator at 4 °C for 16–17 min. Positive results were indicated by the formation of the HA sheet in the wells containing PPRV and negative results were indicated by the formation of the sharp red button of sediment RBCs at the bottom of the well. Red blood cells control appeared as a sharp red button (Figure 2). The end-point dilution for the haemagglutinating virus, which is equal to one HA unit (1 HAU), was determined as the last well that showed complete HA of RBCs. The haemagglutinationtitre (HA titre) of the virus in the sample was determined as the reciprocal of the end-point dilution and expressed as HAU.

### Ethical consideration

This article followed all ethical standards for research without direct contact with human or live animal.

## Results

To investigate the presence of PPR in sheep and goats pneumonic lungs from AL Hasaheisa slaughterhouse, the HA test for the detection of PPRV antigen was performed on 20% lung tissue homogenates prepared from the lung samples. The HA results took 16–17 min to appear, and the elution phenomenon happens rapidly at 18 min post-incubation, where all positive HA results turned negative. With these optimised conditions (PBS with pH 6.8, using V-shaped bottom HA plates and incubation at 4 °C), the HA test could detect PPRV antigen.

**FIGURE 3:**
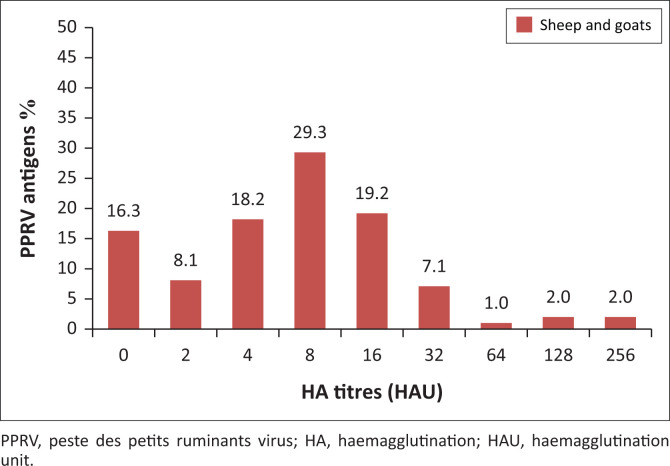
Detection of PPRV in pneumonic lungs of both sheep and goat collected from Al-Hasaheisa slaughterhouse, Sudan. Distribution of haemagglutination titers.

Results of the HA test revealed that 86/99 lung samples were found positive, with an overall higher antigenic prevalence of 86.9%, whilst 13 (13.1%) samples were found negative. Moreover, the HA titres achieved ranged from 2 to 256 HAU, with a mean titre of 17. Moreover, 2 (2.0%) samples achieved the highest HA titre of 256 HAU and 2 (2.0%) samples achieved the highest HA titre of 128 HAU. Apparently, most of the samples (29/99, 29.3%) achieved a titre of 8 HAU (Figure 3).

**FIGURE 4:**
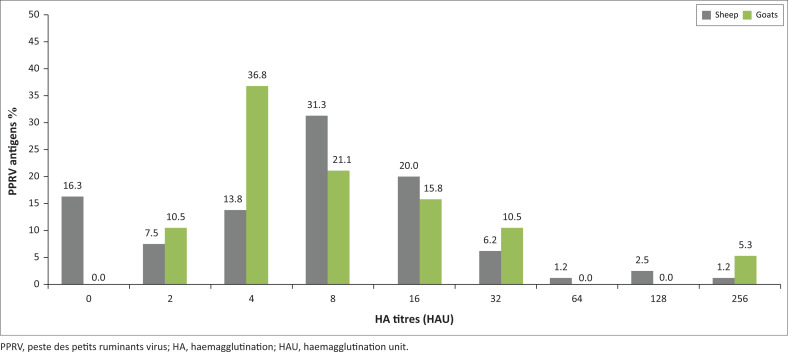
Detection of PPRV in pneumonic lungs of sheep or goat collected from Al-Hasaheisa slaughterhouse, Sudan. Distribution of haemagglutination titers.

Considering the animals’ species under study, in goats, all samples tested (100%, 19/19) were found positive, whereas in sheep, of the 80 tested lung samples, 67 (83.7%) proved positive, whereas 13 (16.3%) lung samples proved negative (Figure 4). Amongst goats, HA results revealed that HA titres ranged from 2 to 256 HAU with a mean titre of 22.7, one sample only (5.3%) achieved the highest HA titre of 256 HAU, whereas most of the samples (7/19, 36.8%) achieved a titre of 4 HAU (Figure 4). Amongst sheep, HA results similarly revealed that HA titres ranged from 2 to 256 HAU with a mean titre of 15.6, one sample only (1.2%) achieved the highest HA titre of 256 HAU and 2 (2.5%) samples achieved the highest HA titre of 128 HAU, whereas most of the samples (25/67, 31.3%) achieved a titre of 8 HAU (Figure 4).

## Discussion

This study was performed to investigate the presence of PPR and further demonstrate its prevalence in pneumonic lung samples from clinically apparently healthy sheep and goats slaughtered at Al-Hasaheisa slaughterhouse, Gezira state, central Sudan. Ninety-nine pneumonic lung samples from sheep (80) and goats (19) were screened using the HA test for detection of PPRV antigen. In this study, the HA test was used because of the demonstrated HA properties of PPRV as stated earlier (Ezeibe et al. 2004; Ramachandran et al. 1993; Wosu 1985, 1991). In a recent study by Rahman et al. (2020), the HA test was proved to be as sensitive as an immuno-capture enzyme-linked immunosorbent assay (IC-ELISA) for detection of PPRV in nasal swabs and faecal samples from clinically PPR-suspected camels in Pakistan.

A 20% lung tissue homogenates were prepared from sheep and goats pneumonic lung samples and used as an antigen in the HA test as performed earlier by Ezeibe et al. (2004) and Osman et al. (2008). Chicken RBCs were used in the HA test because it is the most sensitive RBCs for detection of PPRV antigen and it causes highest HA titers in a relatively short time as documented by Ezeibe et al. (2004) and Osman et al. (2008). Red blood cells suspension with 0.8% concentration in PBS (pH 6.8) was used for obtaining the optimal result of the HA test for PPRV, according to Ezeibe et al. (2004). It is known that virus members of Paramyxoviridae and Orthomyxoviridae cause HA of RBCs and the results were obtained after using the normal conditions of the HA test (PBS with pH 7.4, U-shaped bottom HA plates and incubation at room temperature 20 °C – 25 °C). In this study, using these optimised conditions (PBS with pH 6.8, using V-shaped bottom HA plates and incubation at 4 °C), the HA test could detect only PPRV but not other viruses.

Results of the HA test indicated 86/99 positive samples with an overall higher antigenic prevalence of 86.9%. This indicated the wide distribution of PPR in the central part of the country, which might be affected by the animal movement in these areas. Another study conducted during 2002–2004 in Sudan, using the HA test, revealed higher prevalence (92.5%) amongst sheep and goats than that obtained in this study (Osman 2005; Osman et al. 2008). The prevalence of PPRV antigen obtained from Gezira state in this study is much higher compared to 54.3% prevalence in sheep and goats lung tissue samples, using IC-ELISA, reported recently in the central states of Sudan (Saeed et al. 2017). These results appear much higher compared to 15% antigenic prevalence demonstrated in pneumonic lung samples from sheep and goats, using reveres passive haemagglutination test (rPHA), in White Nile state in Sudan during 2008–2009 (Ishag, Saeed & Ali 2015). Also it is much higher compared to 42.6% prevalence demonstrated in sheep tissue samples from PPR-suspected outbreaks in Sudan during 2008 using IC-ELISA (Saeed et al. 2010). In a recent study, lower prevalence (18.3%) of PPRV antigen was demonstrated in lung samples from different animal species using IC-ELISA (Saeed et al. 2017). In this study, the reported increasing prevalence of PPR is an indication of exposure of these animals to the virus and might be because of insufficient vaccination of animals.

Considering the species basis, all the goat samples (19/19 samples) were found positive with an overall prevalence of 100%. The prevalence of PPRV antigen obtained from Gezira state in this study is much higher compared to 66.8% prevalence reported recently amongst goats in the central states of Sudan (Saeed et al. 2017). These results appear much higher to that described recently by Saeed et al. (2017), who reported a prevalence of 21.1% in lung samples from clinically apparently healthy goats in Sudan using IC-ELISA. In another study, a lower overall prevalence (63%) of PPRV was demonstrated amongst goats in Eritrea by using an immunofluorescence test (Sumption et al. 1988). Another study performed on pneumonic lungs of goats in Bitlis and Van slaughterhouses, Turkey, revealed 40% (17/42) prevalence of PPRV antigen amongst goats by using an immunohistochemical test (IHC) (Yener et al. 2004), which is much lower than the prevalence achieved in this study.

Considering the species basis, 67/80 of the sheep samples were positive with an overall antigenic prevalence of 83.7%. The prevalence of PPRV antigen obtained from Gezira state in this study is much higher compared to 70.7% prevalence reported recently amongst sheep in the central states of Sudan (Saeed et al. 2017). These results appear much higher to that described recently by Saeed et al. (2017), who reported a prevalence of 15.4% in lung samples from clinically apparently healthy sheep in Sudan using IC-ELISA. A much lower antigenic prevalence of 42.6% was demonstrated in sheep, using IC-ELISA, during 2008 in Sudan (Saeed et al. 2010). In the same study conducted in 2008, a lower overall prevalence of 27.3% was demonstrated amongst sheep in Gezira state using IC-ELISA (Saeed et al. 2010).

The findings indicated the presence and the higher prevalence of PPR amongst sheep and goat populations in Al-Hasaheisa Gezira state, Sudan, considering that these lung samples were collected from clinically apparently healthy animals. Detection of PPRV antigen in the pneumonic lung samples is an indication of exposure of these animals to PPRV or PPR viral infection and demonstrates the role of PPR as the cause of pneumonia in small ruminants. In fact, the circulation of the virus in clinically apparently healthy animals, that might excrete the virus in the environment, poses a threat for other in-contact susceptible animals and could play a significant role in the spread of the disease. Results of the study demonstrated the widespread of the disease not only in Al-Hasaheisa town but also in its neighbouring villages because most of these animals found in Al-Hasaheisa were brought from the neighbouring villages. The higher prevalence achieved indicated the potential exposure of these animals to PPRV and the wide distribution of the disease amongst small ruminants in central Sudan, which is considered as PPR endemic areas. For control and rapid elimination of the disease, local authorities should increase the awareness of small ruminant owners, regarding the economic problem and losses caused by the disease and the importance of animal vaccination against the disease.
